# Written in chromatin: The enduring legacy of C. David Allis

**DOI:** 10.1016/j.jbc.2025.110240

**Published:** 2025-07-02

**Authors:** Brian D. Strahl, Gang Greg Wang

**Affiliations:** 1 Department of Biochemistry and Biophysics, University of North Carolina at Chapel Hill School of Medicine, Chapel Hill, North Carolina, USA; 2 Department of Pharmacology and Cancer Biology, Duke University School of Medicine, Durham, North Carolina, USA

“Every amino acid matters… but people matter more.”

C. David Allis

C. David Allis was a visionary scientist whose contributions redefined our fundamental understanding of chromatin. He was also a fantastic mentor whose generosity and unparalleled enthusiasm transformed the lives of all those around him and those who knew him. With his unexpected passing in 2023, the chromatin community lost not only a pioneer but a deeply beloved mentor, collaborator, and friend. To honor Dave’s legacy, we invited contributions from those who worked alongside him and/or were shaped by his vision and mentorship. The result is this special issue of the *Journal of Biological Chemistry*, which brings together reviews from former trainees and colleagues who knew David well and were forever changed by his mentorship and guidance. Each contribution reflects a unique thread of Dave’s influence, illustrating how his intellectual and personal imprint continues to guide the field.

Dave’s career was marked by a series of fundamental discoveries and conceptual breakthroughs that helped elevate chromatin from a boring and static structure to a dynamic regulatory platform that contributed to every aspect of genome function. His early studies in *Tetrahymena*, a single-celled freshwater ciliate, led to the discovery of histone variants and post-translational modifications (PTMs) that could influence gene expression. This model organism was also critical to his landmark breakthrough and discovery of the first transcription-linked histone acetyltransferase, Gcn5, thereby ushering in a new area where chromatin and gene expression were one intimately connected topic. He helped define and uncover the histone-modifier enzyme and effector families—writers, readers, and erasers—that operate on chromatin and proposed the Histone Code Hypothesis, an original intellectual framework that galvanized the field into new thinking about how combinations of PTMs can contribute to specific genome-regulatory outcomes. This idea helped establish the current understanding of how histone PTMs contribute to epigenetics, development, and disease.

In this collection, we sought to reflect the depth and breadth of Dave’s scientific legacy by inviting reviews that touch on major themes in histone biology and chromatin regulation. Sandra Hake and colleagues revisit H2A.Z, the histone variant that Dave first identified as hv1 in *Tetrahymena*, and provide an update on its roles in gene regulation, development, and disease. Tom Muir explores how chemical biology has been applied to chromatin, revisiting the concept of “designer chromatin” he co-developed with Dave to dissect the logic of histone modifications in biology. Yali Dou and Jayme Ogino focus on KMT2A (MLL1), a histone methyltransferase first linked to H3K4 methylation by Dave’s lab and others, and review its structural, functional, and oncogenic roles. Sharon Dent traces the discovery and evolution of lysine acetyltransferases like Gcn5, another milestone from the Allis lab, with a focus on their regulatory complexes, SAGA and ATAC. Chloe Azadegan, John Santoro, and Johnathan Whetstine examine the epigenetic control of DNA amplifications—including extrachromosomal elements—linking chromatin dynamics to emerging mechanisms of tumor adaptation and therapeutic resistance. Finally, Elizabeth Duncan revisits the phenomenon of histone tail clipping, blending a scientific review of this enigmatic process with a personal narrative of discovering it as a graduate student in Dave’s lab—offering a deeply reflective account of both the science and the mentorship that shaped it.

Together, the reviews in this issue highlight the continuing impact of Dave’s work and the fertile directions it has inspired. They span model systems and disease contexts, experimental approaches, and conceptual frameworks. And while they cover distinct topics, each is rooted in a central idea that Dave championed: that chromatin is not merely a structural element of the genome, but a dynamic player with exquisite genome-regulatory functions. It carries a language—encoded by histone PTMs, histone variants, and many other chromatin-associated proteins and enzymes—that functions like a symphony to orchestrate the complex events in chromatin.

We are grateful to the authors for contributing their insight and scholarship, and to the *JBC* editorial team for supporting this commemorative issue. Dave once said, “Every amino acid matters… but people matter more.” His science—and his life—embodied that ethos. We hope this tribute honors both.

